# Reconciling concepts of black queen and tragedy of the commons in simulated bulk soil and rhizosphere prokaryote communities

**DOI:** 10.3389/fmicb.2022.969784

**Published:** 2022-09-15

**Authors:** Damien Robert Finn, Mario App, Lionel Hertzog, Christoph C. Tebbe

**Affiliations:** Thünen Institut für Biodiversität, Johann Heinrich von Thünen Institut, Braunschweig, Germany

**Keywords:** ecological modelling, bacterial interactions, terrestrial microbiology, bacterial evolution, differential equation

## Abstract

The Black Queen hypothesis describes the evolutionary strategy to lose costly functions in favour of improving growth efficiency. This results in mutants (cheaters) becoming obligately dependent upon a provider (black queen) to produce a necessary resource. Previous analyses demonstrate black queens and cheaters reach a state of equilibrium in pair-wise systems. However, in complex communities, accumulation of cheaters likely poses a serious burden on shared resources. This should result in a Tragedy of the Commons (ToC), whereby over-utilisation of public resources risks making them growth-limiting. With a collection of differential equations, microbial communities composed of twenty prokaryote ‘species’ either from rhizosphere, characterised by abundant carbon and energy sources, or bulk soil, with limited carbon and energy supply, were simulated. Functional trait groups differed based on combinations of cellulase and amino acid production, growth and resource uptake. Randomly generated communities were thus composed of species that acted as cellulolytic prototrophic black queens, groups that were either cellulolytic or prototrophic, or non-cellulolytic auxotrophic cheaters. Groups could evolve to lose functions over time. Biomass production and biodiversity were tracked in 8,000 Monte Carlo simulations over 500 generations. Bulk soil favoured oligotrophic co-operative communities where biodiversity was positively associated with growth. Rhizosphere favoured copiotrophic cheaters. The most successful functional group across both environments was neither black queens nor cheaters, but those that balanced providing an essential growth-limiting function at a relatively low maintenance cost. Accumulation of loss of function mutants in bulk soil risked resulting in loss of cumulative growth by ToC, while cumulative growth increased in the rhizosphere. In the bulk soil, oligotrophic adaptations assisted species in avoiding extinction. This demonstrated that loss of function by mutation is a successful evolutionary strategy in host-associated and/or resource-rich environments, but poses a risk to communities that must co-operate with each other for mutual co-existence. It was concluded that microbial communities must follow different evolutionary and community assembly strategies in bulk soil versus rhizosphere, with bulk soil communities more dependent on traits that promote co-operative interactions between microbial species.

## Introduction

A notable aspect of soil microbial communities is inherently high biodiversity. A singular macroaggregate of soil, *circa* 1 mg in weight and 2 mm in diameter, can be home to 2,000 prokaryotic taxa, considered unique sequence variants of a portion of the 16S rRNA gene (Szoboszlay and Tebbe, 2021). Undoubtedly, spatial heterogeneity not only at the macroaggregate scale but also within the microaggregates that comprise these structures (Tisdall and Oades, 1982) support the formation of oxygen, nutrient and pH gradients that foster a wide range of potentially habitable niches (Rillig et al., 2017; Wilpiszeski et al., 2019). Diverse potential of niches begets biodiverse communities (Hutchinson, 1957; Leibold, 1995). Another factor contributing to biodiversity are ecological interactions between taxa necessary for their mutual survival (Amor and Bello, 2019). For example, singular soil microbial taxa do not typically catabolise all chemical structures within complex plant material, but rather the labour is divided amongst different ecologically distinct functional groups. An archetypal example in soil processes is the successional, preferential breakdown of soluble, non-lignified carbohydrates and lignin by taxa such as *Alternaria*, *Cladosporium* and white rot fungi, respectively (Køller and Struwe, 2002; Vivelo and Bhatnagar, 2019). Bacteria divide the labour of essential growth metabolite production. For example, 76% of 949 free-living, gut commensal and endosymbiotic bacterial genomes are auxotrophic for at least one metabolite and thus must share the burden of production between a host and/or other microbial community members (D’Souza et al., 2014).

While two microbial taxa that co-operate with each other can be more productive than either growing alone (Pande et al., 2014), (micro)organisms that form co-operative relationships put themselves at risk of ‘defectors’ or ‘cheaters’ (Nowak, 2006). Cheaters can be considered as (micro)organisms that do not pay the costs associated with co-operation, e.g. growth metabolite production, while reaping the rewards of others’ labour (Nowak, 2006). This is a successful evolutionary strategy for optimizing growth efficiency *via* the loss of genome-encoded, costly enzymatic/functional machinery, termed ‘genome streamlining’ (Mira et al., 2001). Selection-driven loss of function is demonstrated succinctly by *Escherichia coli* grown successively under conditions where glucose is the sole available carbon source—over several thousand generations *E. coli* loses the capacity to catabolise a broad range of saccharides, amino acids and fatty acids (Cooper and Lenski, 2000). Similarly, when grown in the presence of amino acids, auxotrophic *E. coil* mutants develop (D’Souza and Kost, 2016). Some of the Earth’s most abundant Bacteria, such as *Pelagibacter ubique* and *Prochlorococcus*, exemplify the successful adaptation of living with a streamlined genome (Giovannoni et al., 2005, 2014).

Genome reduction towards a cheater lifestyle is only successful where another (micro)organism continues to provide the lost essential growth factor(s). In microbial ecology, the evolutionary race to lose functions has been termed the Black Queen hypothesis, as something of a corollary to the more classical evolutionary Red Queen hypothesis associated with function gain (Morris et al., 2012). Here, the black queens are the taxa that have been too slow to lose a function, and are forced to carry the burden of production for the mutual survival of the microbial community. Meanwhile, the cheaters can preferentially invest carbon and energy into more efficient reproduction at the ultimate cost of their population size being obligately dependent upon, and thus constrained by, the work of the black queen. In simulated (Mas et al., 2016) and *in vitro* (Morris et al., 2014) two-species systems, black queen and cheater populations coexist in a state of equilibrium. These principles apply to a range of essential products and services in soil microbial communities, including nitrogen fixation, cellulase, siderophores, amino acid and vitamins (Berlemont and Martiny, 2013; D’Souza et al., 2014; Morris, 2015; Butaitė et al., 2017). These products/services are also termed ‘public goods’. In reality, most taxa likely do not exist as ‘true’ black queens that carry out all possible functions, or cheaters that carry out none, but rather as niche differentiated intermediates that rely on mutualistic interactions with others that complement their own functions (Amor and Bello, 2019). While the simulations performed here are glaringly simplistic relative to reality (discussed further below) the binary terms for black queen and cheater are used to refer to taxa that carry the relatively greatest and least burdens for production of public goods, respectively.

Becoming dependent on a public good poses its own risks, particularly if it must be shared amongst others. Hardin (1998) eloquently explained the “Tragedy of the Commons” in terms of the over-utilisation of a shared resource by individual humans within a society. The premise is that, where the benefit of an individual taking a finite resource from the common public good is *x* but the cost shared amongst *y* individuals is *x*/*y*, each individual is driven to take *x*. From the individual’s perspective, the cost disadvantage is minimal relative to the benefit, and so there is no incentive for the individual to relax demand. The loss of the resource is inevitable where the public good is finite. In regard to microbial taxa within a community, as cheaters for a given resource emerge over time, and grow more efficiently relative to the black queen that continues to produce the resource, there will be an increasing demand of *y* on a finite *x*.

Unlike humans, microorganisms have had over 3.5 billion years to learn how to coexist within communities. This raises the question of how microbial taxa have developed to persist while genome streamlining is eternally producing cheaters with the potential of driving communities towards a Tragedy of the Commons. As all biological systems are at potential risk, it is not unreasonable to expect extant (micro)organisms to have evolved mechanisms to prevent or at least reduce the severity of a Tragedy outcome (Rankin et al., 2007). It has been considered that Tragedies are avoided because black queens preferentially benefit from their own public goods (Mas et al., 2016). While this would certainly hold true for goods such as amino acids, it is difficult to see how it could consistently apply to secreted enzymes that catalyse extracellular reactions, such as cellulase. If a cellulolytic taxon cannot acquire sufficient di- and mono-saccharides to support its growth, it will cease to produce cellulase and, without steady production, any cheaters dependent on its activity will perish. Even in saccharide-producing reactions localised to a cell’s periplasmic space, e.g. invertase activity in yeast, *circa* 99% of the produced glucose can be lost to neighbouring, potential cheater cells (Gore et al., 2009). Furthermore, theoretical and experimental work have primarily considered coexistence in two-species systems (Morris et al., 2014; Pande et al., 2014; Mas et al., 2016) which is probably not the norm in most soil habitats. Where interactions between more than two species have been modelled, community stability is threatened where non-mutualistic resource cross-feeding (i.e. black queen to cheater) prevails over mutualistic, division of labour sharing of resources between different taxa (Butler and O’Dwyer, 2018). As long as at least one taxon capable of producing the required public good persists, then the growth of any dependent taxa will continue but be limited by it. This will not result in system collapse *per se*, but rather restricted growth of individuals, and collectively, the community. Thus, here a Tragedy outcome refers to a community whose collective growth suffers due to an over-abundance of taxa that do not contribute to public good production.

To gain insight into how communities may avoid a Tragedy outcome, a mathematical simulation of a soil aggregate microbial community was developed, with 20 ‘species’ placed into two ‘environments’. In the bulk soil environment, species were forced to compete and/or co-operate with each other to acquire glucose derived from cellulose catabolism whilst producing (or taking) amino acids. This gave rise to four potential functional trait groups: cellulolytic prototrophs (black queens), cellulolytic auxotrophs, non-cellulolytic prototrophs (both groups that pay intermediate costs of public good production) and non-cellulolytic auxotrophs (cheaters). These traits were chosen as both are examples of public goods that play a role in community assembly (Morris, 2015), with cellulase as an example of a secreted hydrolytic enzyme necessary for generating a growth substrate from complex polymers (Gore et al., 2009) and amino acid production as an example of sharing essential growth factors to auxotrophs incapable of synthesising their own (D’Souza and Kost, 2016). In the rhizosphere environment, a constant supply of glucose and amino acids was provided to species over time akin to plant root exudates. The rhizosphere environment had the same potential functional groups as the bulk soil. Under both scenarios, species could evolve to lose cellulolytic and amino acid production functions over time. Finally, overlaying functional trait combinations were physiological traits associated with canonical copiotrophic (high maximum growth rate, low transporter affinity) or oligotrophic (low maximum growth rate, high transporter affinity) life strategies (Koch, 2001). The combination of functional trait groups and life strategy gave rise to eight possible distinct profiles. We specifically sought to address the following: (1) In communities with mixed functional traits, what was the overall relationship between biodiversity and biomass production? (2) What was the specific contribution of each functional group to community biomass production? (3) Did the accumulation of cheater species over time (i.e. by speeding up or slowing down rate of functional trait loss) drive communities towards a Tragedy outcome? and (4) Could Tragedies be avoided through physiological adaptations unrelated to public goods? Herein species are considered as individuals, while functional groups are considered as all species that share a combination of traits (e.g. cellulolytic prototrophs).

## The model

**Figure 1:**
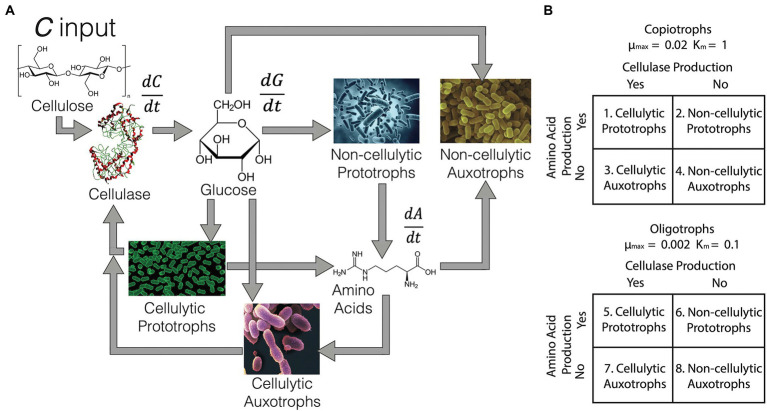
**(A)** General outline of the bulk soil environment model. Equations for change in cellulose (*C*), glucose (*G*) and amino acids (*A*) are outlined in Supplementary Equations 1–5. Briefly, *C* enters the system and is degraded by cellulase produced by cellulolytic species. Catabolised *C* produces *G,* which acts as a common resource for all species. In addition, prototrophic species produce *A*, which is taken up by auxotrophs. Black queens are equivalent to cellulolytic prototrophs, and cheaters as non-cellulolytic auxotrophs. **(B)** Interaction between life strategies (as copiotroph or oligotroph) and functional trait combinations. The maximum growth rate (μ_max_) and transporter affinity (K_m_) for each life strategy is noted. There are eight possible combinations of life strategy and functional group which were randomly generated for each ‘species’ in each simulated community (species *n* = 20 per community).

Figure 1A is a conceptual diagram of the interactions between different functional groups and resource pools (cellulose, glucose and amino acids) in the bulk soil environment. Here, 20 ‘species’ of eight potential randomly generated trait and life strategy combinations (Figure 1B) were placed together and given 2 mM of cellulose every 100 generations for 500 generations. The periodicity of the cellulose pulse was determined during model optimisation to prevent extinction of all species in the system. No glucose or amino acids were provided to bulk soil communities, and thus the taxa were forced to produce their own. Twenty randomly generated species were also placed in a rhizosphere environment, where 2 mM glucose was provided at every generation over 500 generations and differential equations were altered to make amino acids non-limiting. The concentration and input rate of glucose was also based on the minimum input required to prevent extinction of all species, based on model optimisation. Therefore, in the rhizosphere environment, species did not need to produce their own glucose or amino acids. Each randomly generated species had an equal probability of being one of the four functional groups, and an equal probability of being one of the two life strategies. Cumulative biomass production and Shannon alpha-diversity index were tracked for each simulation. Differential equations for changes in resource and species’ biomass over time are given as Supplementary Equations 1–5. Inspiration for the differential equations was originally drawn from Vet et al. (2018), in addition to classical Monod, Michaelis–Menten and non-linear bacterial growth dynamics (Kayser et al., 2005). Specifically, Monod kinetics were used to describe the leakage of amino acids from prototrophs to the environmental pool. Michaelis–Menten kinetics described cellulase activity and membrane transporter uptake of glucose and (for auxotrophs) amino acids. Oligotroph transporters had a higher affinity for resources than copiotrophs. Bacterial growth was dependent on a species’ maximum growth rate, growth efficiency on glucose (based on continuous culturing of *E. coli*, Kayser et al., 2005) and the maintenance burden associated with a given set of traits, discussed further below. Copiotrophs had an order of magnitude higher maximum growth rate than oligotrophs. In-depth explanations of all model parameters are given in Supplementary Table 1. Model parameters for cellulase, amino acid production, transporters and growth rates were taken from the literature (Supplementary Table 1). All R code to reproduce the model is available at: github.com/DamienFinn/Black_Queen_simulations.

The LSODA solver (Hindmarsh, 1983; Petzold, 1983), applying the ‘ode’ function from the package ‘deSolve’ (Soetaert et al., 2010), was used to compute changes in resource pools and species biomass over time. The ‘diversity’ function from the package ‘vegan’ was used to calculate Shannon index (Oksanen et al., 2013). Supplementary Figure 1 is an example of a growth curve depicting biomass of a copiotrophic cellulolytic prototroph, cellulose and glucose pools over 200 generations. The species shows initial exponential growth, followed by a stationary phase and finally death once glucose is reduced below 10 μM.

A critical aspect of the Black Queen hypothesis and the model employed here relates to a species’ maintenance burden. Genome streamlining results in the loss of expensive functional traits, thereby reducing a species’ maintenance burden. Those species with relatively low maintenance burden grow (and compete for resources) more efficiently than their encumbered neighbours. It was assumed that cellulase was more costly to maintain and to express than amino acid production. Thus, the relative burdens were aligned as: black queens > cellulolytic auxotrophs > non-cellulolytic prototrophs > cheaters. Furthermore, the maintenance burden of oligotrophs was considered slightly greater than copiotrophs as high-affinity membrane transporters are energy dependent (i.e. ATP-binding cassette transporters; Davidson and Chen, 2004).

With each generation, within each simulation, there was a random chance for a loss of function mutation to occur within each species. Scenarios for four different mutation rates were run at: null (0 per generation), low (4.5 × 10^−4^), medium (4.5 × 10^−3^) and high (4.5 × 10^−2^) rates. The medium value was based on observed mutation rates per generation in *E. coli* (Cooper and Lenski, 2000). It is acknowledged that mutation rates in *E. coli* will not be reflective of many soil taxa, and rather results from these simulations should be considered in relative rather than absolute terms. This gave rise to eight distinct scenarios, with combinations of two environments (bulk soil and rhizosphere) and four mutation rates (null, low, medium and high).

The following is a brief description of how each scenario was performed using the bulk soil environment and null mutation rate scenario as an example. In Simulation 1, 20 species were randomly generated and placed within the bulk soil environment to grow over 500 generations, where each species had a rate of 0 loss of function mutations per generation. In Simulation 2 of the bulk soil null mutation scenario, a completely new composition of 20 random species was generated and placed within the bulk soil environment to grow over 500 generations, where each species had a rate of 0 loss of function mutations per generation, and so on. One thousand Monte Carlo simulations were performed for each environment and mutation rate combination. In total, 8,000 simulations were run.

It was assumed that other essential resources for growth (e.g. oxygen, nitrogen, phosphorous) were non-limiting. Space was not incorporated into the model. Rather, it was assumed that these relatively small communities, consisting of only 20 species, coexist within a soil aggregate at a proximity and moisture content where Monod and Michaelis–Menten kinetics could explain resource uptake. This was intended to fall within the modelling framework of Raynaud and Nunan (2014) that suggests only 2–100 bacterial species can realistically be considered ‘neighbors’ within 10 μm of each other. Finally, it was assumed that prototrophs overproduce and leak amino acids to the environment rather than strictly regulate production for their own needs and/or have mechanisms to prevent leakage. Potential limitations of these assumptions are discussed further below.

## Do diverse communities function better?

If the loss of functions can negatively affect community growth, then possessing a diverse set of functions must therefore be beneficial. To this end, a positive relationship between biodiversity (as Shannon index) and community function (as cumulative biomass production) was investigated. Positive relationships between microbial biodiversity and terrestrial ecosystem function have been demonstrated (Wagg et al., 2014; Delgado-Baquerizo et al., 2016) although much of this may be redundant in that if multiple taxa perform the same function then the loss of one does not necessarily impact the rate of an ecosystem process (Griffiths et al., 2000; Wertz et al., 2007; Allison and Martiny, 2008). This was tested under the bulk soil and rhizosphere null mutation rate scenarios. Without loss of function mutations, the traits of species remained fixed. That is, within each simulation, the functional profile (e.g. cellulolytic prototroph, non-cellulolytic auxotroph) of each randomly generated species did not change. In this way, the null mutation scenarios could be considered as a sort of positive control not at risk of accumulating cheaters. The Shannon index, which is a function of both community Richness and Evenness (Hill, 1973), was measured at the final point of the simulations. Where a high number of species survived with relatively even biomass, the Shannon index was relatively high. Where species went extinct or biomass became uneven, for example if some species were markedly more successful than others, the Shannon index was low. The success (or lack thereof) of each community was therefore a consequence of the functional traits possessed across species.

**Figure 2:**
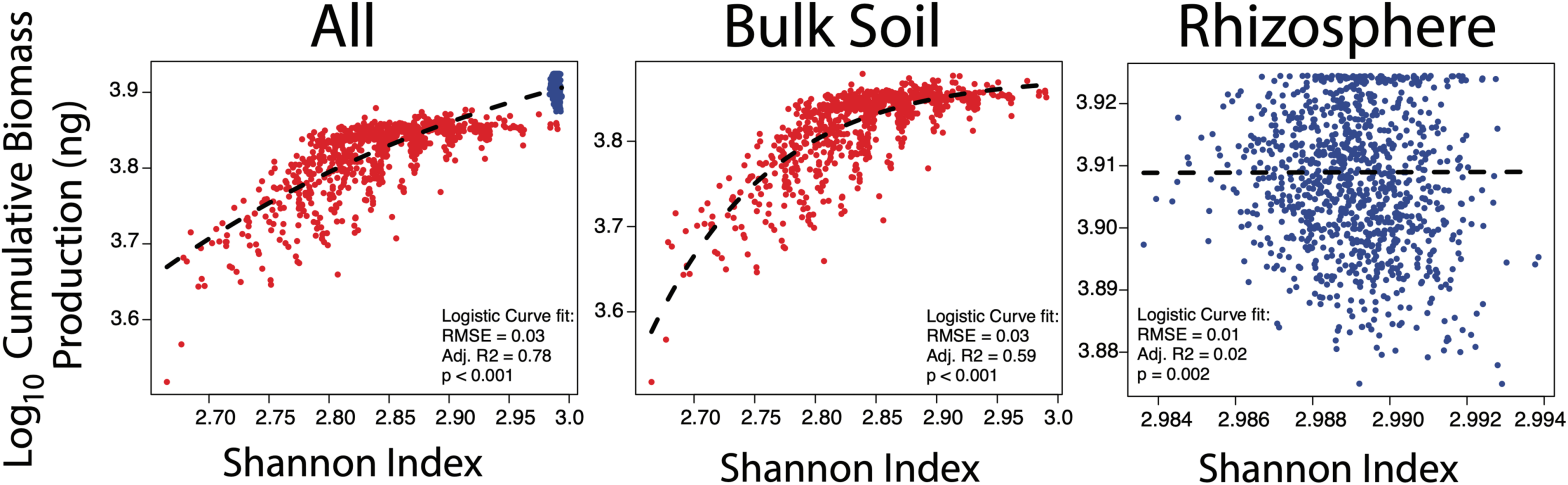
Relationship between biodiversity (Shannon index) and biomass production in all null model simulations (*n* = 2,000), the bulk soil simulations (*n* = 1,000) and the rhizosphere simulations (*n* = 1,000). Each point represents the cumulative biomass (ng, *y*-axis) production of a community of a single simulation against the biodiversity of that community (Shannon index, unitless, *x*-axis) at the end of the simulation at generation 500. Bulk soil simulations are in red and rhizosphere in blue.

The benefit of biodiversity was entirely dependent on the environment the species found themselves in (Figure 2). In the bulk soil, biodiversity showed a positive logistic relationship with cumulative biomass production (Supplementary Equation 6, *R*^2^ = 0.59, *p* < 0.001), with parameters fit *via* non-linear least squares regression ‘nls’ function in *R* (Bates and Watts, 1988) whereby poorly diverse communities performed poorly relative to those with higher biodiversity. However, above a certain threshold (*circa* Shannon of 2.85) an increase in biodiversity was subject to diminishing returns in biomass production. Conversely, when glucose and amino acids were supplied to the rhizosphere community, this relationship dissipated. Despite this loss in relationship, both community properties were significantly greater in the rhizosphere (Supplementary Figure 2; Welch’s *t*-tests *p* < 0.001). This agrees well with community assembly theory, which suggests environments that lack a strong filter are likely to be more forgiving in who persists there, resulting in stochastic assemblages (Dini-Andreote et al., 2015). In our simulations, glucose and amino acid limitation were thus strong determiners for diverse assemblages. In other words, the success of the bulk soil communities was dependent on how effectively the species could co-operate, therefore maximising the potential functional trait repertoire (i.e. biodiversity) was beneficial here. In contrast, these particular traits were of no relevance in the rhizosphere environment as all species were supplied directly with their growth requirements.

These results should not be interpreted as stating biomass production and biodiversity are greater in the rhizosphere relative to bulk soil, or that these properties are uncoupled in the rhizosphere. The rhizosphere is typically considered to have a lower biodiversity as plant root exudates select for a proportion of responsive taxa sourced from the surrounding bulk soil (Philippot et al., 2013). Furthermore, the decoupling of biodiversity and biomass production is likely a consequence of the traits given to species here. Essential traits for life in the rhizosphere include pili attachment to root tips (Dupuy and Silk, 2016), catabolism of plant mucilage (Benizri et al., 2007), motility and rapid growth (Dennis et al., 2010). Rather, these results suggest that cellulase and amino acid production are redundant in the host-associated rhizosphere. The answer to the first hypothesis was therefore that where functional traits are non-redundant, either due to environmental pressures or other taxa, community growth benefits from biodiversity.

## Are some functional groups more desirable than others?

**Figure 3:**
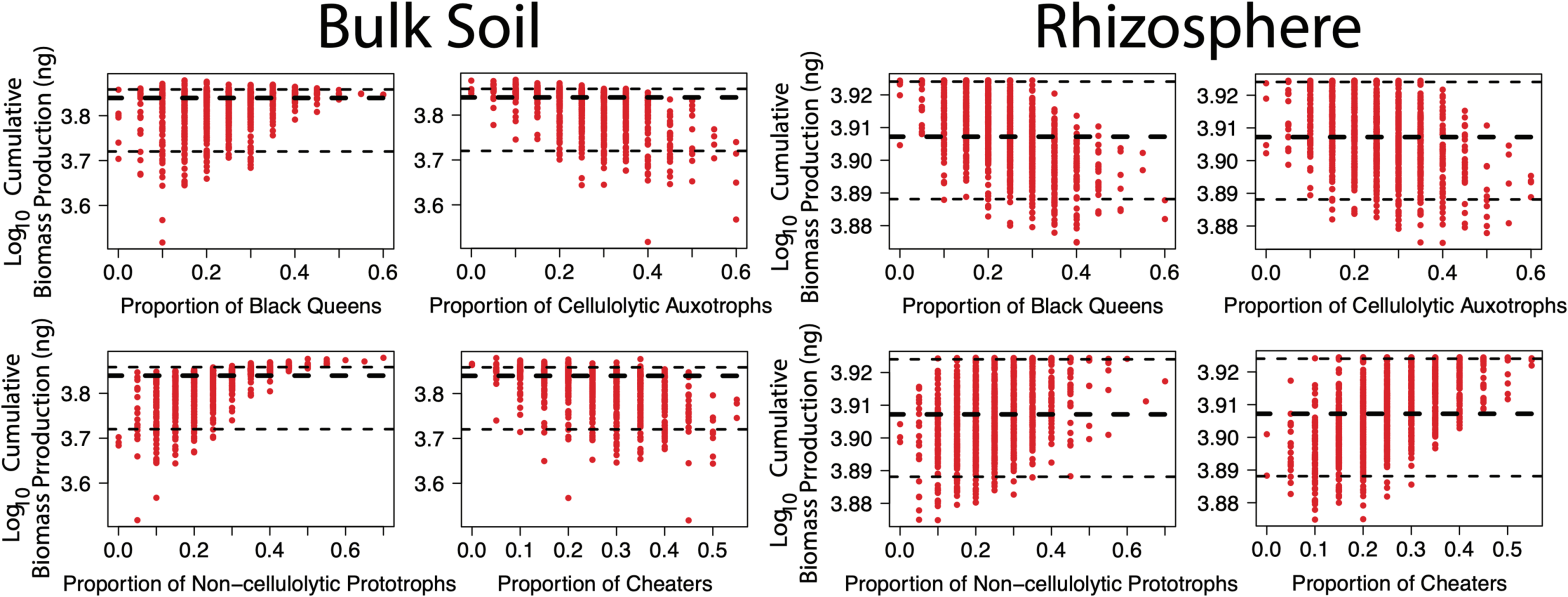
Relationship between functional groups and cumulative biomass production in simulated communities under null mutation rates. The *x*-axis shows relative proportion of a given function within twenty species communities. The *y-*axis shows cumulative biomass production (ng) over 500 generations, per simulation. The thick dotted black lines show median cumulative biomass production, while the thin dotted black lines show upper and lower 5% quantiles. Black queens are equivalent to cellulolytic prototrophs and cheaters as non-cellulolytic auxotrophs.

Hypothesis two sought to evaluate whether certain functional groups were greater contributors towards overall biomass production. As above, this was assessed in the null mutation scenarios. In the bulk soil environment, there was a risk of biomass production being significantly lower where the community was comprised of < 30% black queens and/or < 25% non-cellulolytic prototrophs (Figure 3; lower dotted black line indicating 5% quantile). As the communities had fixed total species numbers, a relatively high proportion of cellulolytic auxotrophs and/or cheaters was the inverse. Interestingly, the non-cellulolytic prototrophs conveyed a relatively greater benefit to the community than the black queens (beta-coefficient 0.27, *R*^2^ = 0.35 versus beta-coefficient 0.16, *R*^2^ = 0.12, respectively, Supplementary Table 2). Indeed, if the community was comprised of over 45% non-cellulolytic prototrophs, biomass was consistently greater than the median and all high-producing communities had over 35% of this functional group (Figure 3; higher dotted black line indicating 95% quantile). These general trends were also observed in how the proportion of each functional group contributed to biodiversity in bulk soil (Supplementary Figure 3).

These results suggest two things: (1) amino acid production had a relatively greater benefit for community growth than cellulase, indicating that amino acids were the growth-limiting factor in the bulk soil scenarios; and (2) relative to black queens, the lower maintenance burden of non-cellulolytic prototrophs allowed them to grow better and produce more amino acids, which had a greater overall benefit for the community. The difference between these two functional groups is a novel observation due to these simulations expanding beyond modelling pairwise, black queen-cheater interactions (Morris et al., 2014; Mas et al., 2016) and agrees with other larger-scale modelling that points to integral roles of taxa that provide growth-limiting factors (Butler and O’Dwyer, 2018). Liebig’s Law of the Minimum dictates that there will always be at least one growth-limiting factor (Danger et al., 2008) and this may change across time and space. *In situ* concentrations of amino acids are lower than major carbon substrates, such as poly-, di- and mono-saccharides (Moe, 2013). However, more carbon is necessary for growth relative to the *circa* 50 to 500 μ moles amino acids per gram dry weight in *E. coli* (Reitzer, 2009) making it difficult to suppose which is likely to be more limiting *in situ*. Many studies on soil organic carbon cycling suggest saccharides are the growth-limiting factor for microbial activity (Fontaine et al., 2007; Kuzyakov and Blagodatskaya, 2015). Nitrogen is also an important growth-limiting factor that was not included in this model. An interesting implication of these simulations is that once sufficient nutrients (e.g. carbon, nitrogen) to support growth are available, then ecological interactions between prototrophs and auxotrophs may become the next factor that limits growth.

An additional consideration on the relationship between black queens and non-cellulolytic prototrophs is that, *in situ*, black queens that retain many functions (relative to other taxa) may not ultimately be that important for community growth. Rather, those taxa that have experienced some function loss to reduce maintenance burdens, yet retain essential functions, would be more desirable. This implies that genome streamlining is greater than a zero-sum game that produces function-less cheaters and instead may act at the community level to improve the efficiency of ecological interactions and overall growth.

The relationship between black queens and cheaters was reversed in the rhizosphere, where having the lowest maintenance burden made cheaters the best contributors to biomass production (Figure 3; Supplementary Table 2). Biodiversity was unaffected by proportionality of functional groups in rhizosphere (Supplementary Figure 3). Increasing proportions of both black queens and cellulolytic auxotrophs reduced biomass production due to the large maintenance burden of cellulase. *In situ* observations of rhizosphere communities tend to show enrichment of fast-growing Beta-, Alphaproteobacteria and glycosyl-transferase depleted actinobacterial Micrococcaceae on root exudates (Li et al., 2014; Ai et al., 2015; Gkarmiri et al., 2017). Conversely, the relative abundance of slow-growing potentially cellulolytic taxa from Acidobacteria and Verrucomicrobia tends to decrease. This effect is typically explained as one group being relatively more copiotrophic than the other. These simulations suggest a taxon’s maintenance burden is a potentially important over-looked contributor to whether it can be considered a copiotroph, with previous studies mostly focused on copy numbers of the 16S rRNA gene (Vieira-Silva and Rocha, 2010), ABC transporters (Lauro et al., 2009) or a combination of traits involved in glutathione, oxidative phosphorylation and pyruvate metabolism (Finn et al., 2021). Worth noting is that genome size (or total number of encoded proteins) alone may not be indicative of maintenance burden, with representatives of the Acidobacteria and Verrucomicrobia being similar in size to fast-growing Proteobacterial Pseudomonadaceae, Rhizobiaceae and Burkholderiaceae (Finn et al., 2021) and maximum growth rate being generally independent of genome size (Westoby et al., 2021). From a practical perspective it would be challenging to reliably predict (or measure) a taxon’s maintenance burden, but even so, considering the *absence* of certain traits (e.g. cellulase) could also assist in explaining copiotroph–oligotroph behaviour.

Finally, worth noting is that non-cellulolytic prototrophs remained effective contributors to biomass production in the rhizosphere, albeit of relatively lesser importance than in bulk soil (Figure 3; Supplementary Table 2). This was despite their role being redundant here. The success of this group in both environments can be explained by having an essential, yet relatively low-maintenance, function. Thus, in regard to hypothesis two, non-cellulolytic prototrophs and cheaters were the main contributors to biomass production in bulk soil and rhizosphere, respectively, however the non-cellulolytic prototrophs were generally efficient contributors.

## Can loss of function cause a tragedy of the commons?

**Figure 4:**
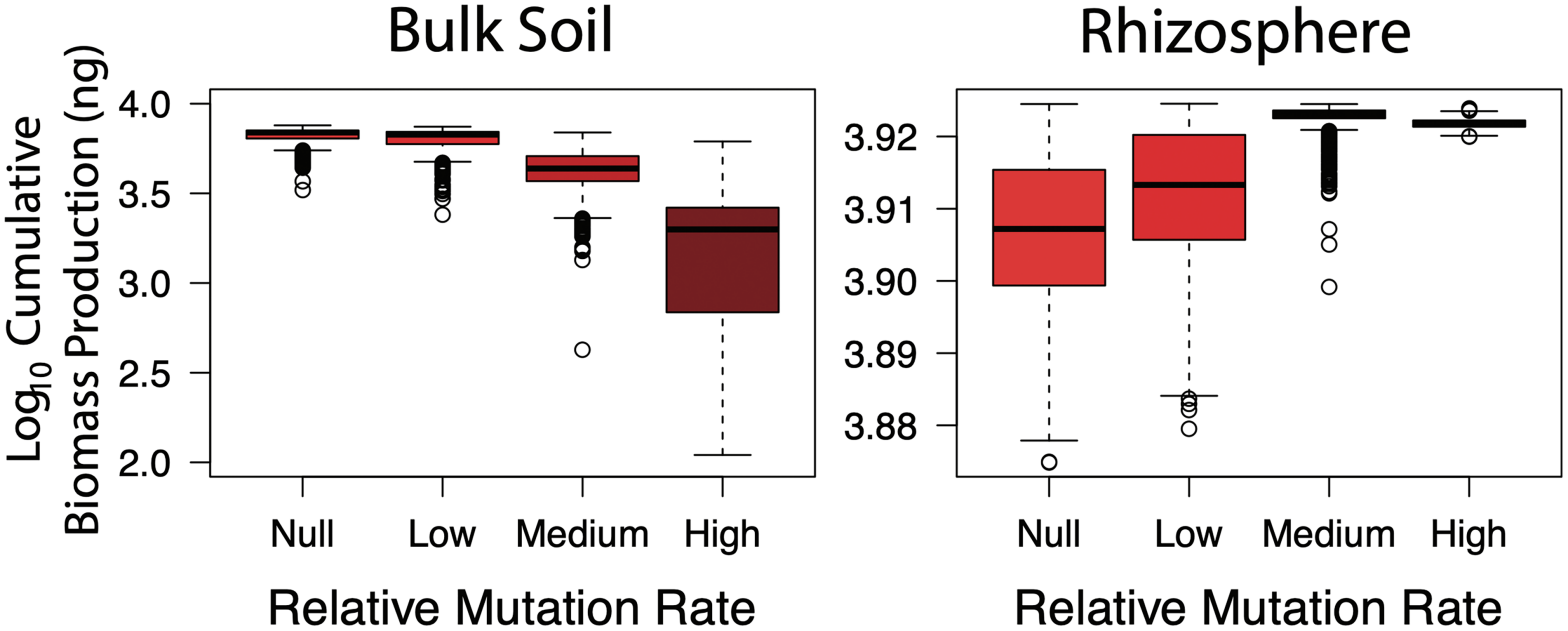
Cumulative biomass production (ng) in simulated bulk soil and rhizosphere communities with increasing rates of loss of function mutations. The higher the rate, loss of function mutants emerge and accumulate earlier in community simulations.

Hypothesis three was concerned with the temporal component of the Black Queen hypothesis, whereby loss of function mutations occurs over time as selection favours taxa with streamlined genomes. To address this, bulk soil and rhizosphere scenarios were conducted under low, medium and high mutation rates. Once again, the impact of loss of function was environment dependent (Figure 4). In the bulk soil, as the rate of function loss increased, biomass production was severely hindered (Kruskal–Wallis, *p* < 0.001). Relative to the null mutation rate, up to 1.5 orders of magnitude (or *circa* 3,000 ng) of biomass productivity was lost under the highest rate. Under the medium rate based on observed loss of function mutation rates in *E. coli* (Cooper and Lenski, 2000) there was a smaller loss of 100–1,000 ng. The loss of potential productivity due to the accumulation of mutants agrees well with the Tragedy of the Commons—as the bulk soil communities lost taxa that produced glucose and amino acid resources, the system was put under strain. However, assuming the medium rate is best reflective of what occurs *in situ*, then most bulk soil communities were still able to function relatively well despite the increased burden.

Conversely, marginal (yet significant) increases in biomass production were observed in the rhizosphere (Kruskal-Wallis, *p* < 0.001), between *circa* log_10_ 3.91 to log_10_ 3.92, or roughly 200 ng. This demonstrated the advantage of genome streamlining, which is commonly observed in host-associated (i.e. commensals and pathogens) taxa (Mira et al., 2001). The combined effect of cellulase and prototrophy being redundant, in addition to the growth benefits of reducing maintenance costs, made function loss the successful strategy in this environment. Therefore, similar to hypothesis one, the negative impact of loss of function mutants putting increased strain on public resources occurred in the bulk soil community which was dependent on cellulase and amino acid production functional traits and co-operative interactions.

## Physiological adaptations to resist tragedies

*Pelagibacter ubique* and *Prochlorococcus* are two well-described examples of both genome streamlining and black queen interactions (Morris et al., 2012; Giovannoni et al., 2014). Specifically, this is because they have lost the capacity to synthesize a range of vitamins and catalase, respectively, which are essential for their growth. Both taxa are therefore dependent on obtaining these public goods from other members of their communities, either as potential cheaters (*P. ubique*) or as co-operative autotrophs that can supply organic matter in exchange for catalase (*Prochlorococcus*).

These taxa are native to nutrient-poor, highly oligotrophic marine surface waters, which is a markedly different environment to a host-associated, rhizosphere-like environment where growth factors are constantly provided. Assuming that adaptations to life under oligotrophic conditions may explain how these taxa survive despite being auxotrophs for essential resources they require, the fourth hypothesis tested the importance of physiological traits for species survival and community biomass production. In practice, this involved altering the maximum growth rate, transporter uptake affinity and maintenance burden to make some species ‘copiotrophs’ and others ‘oligotrophs’.

**Figure 5:**
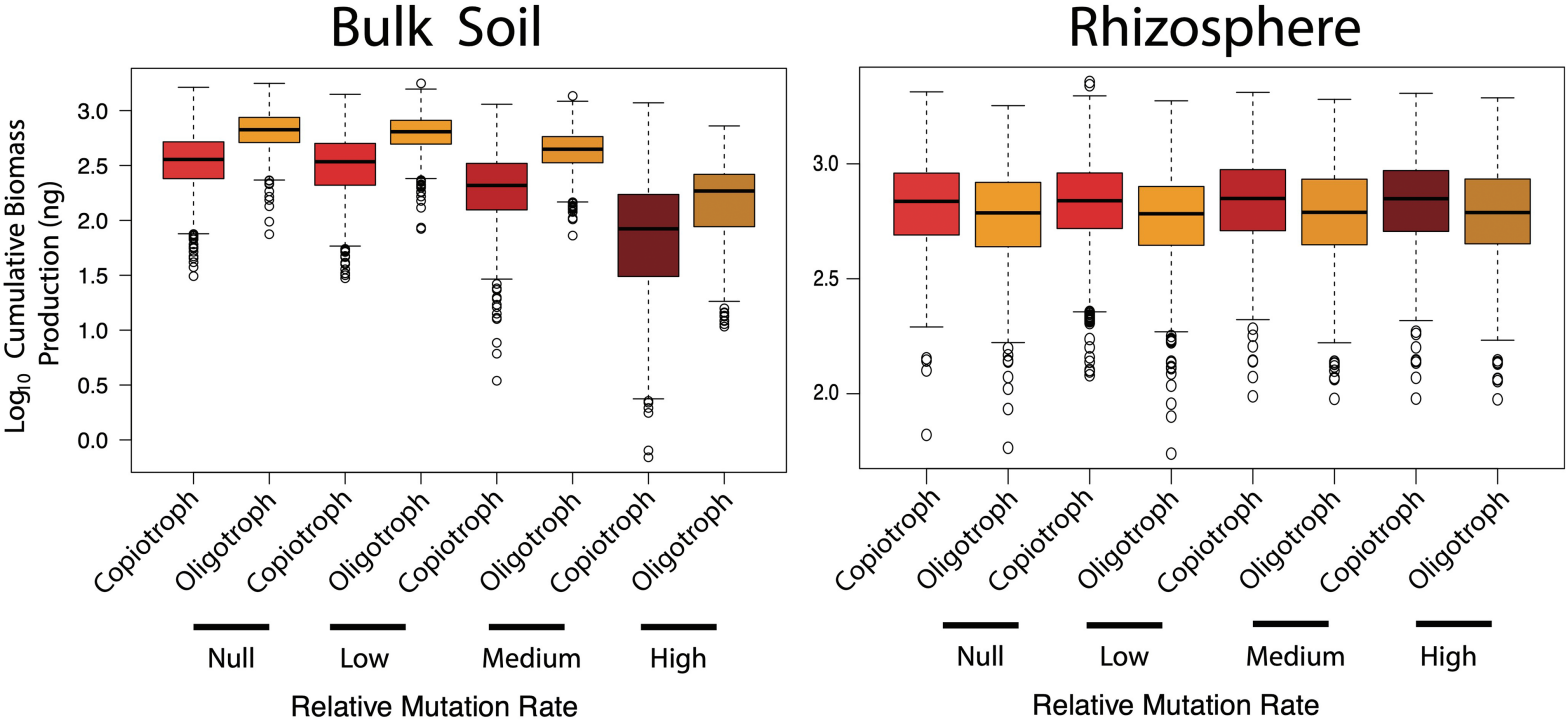
Cumulative biomass production (ng) of copiotrophic and oligotrophic species in simulated bulk soil and rhizosphere communities over differing mutation rates.

In the bulk soil, oligotrophs always produced more biomass than copiotrophs regardless of how many loss of function mutants emerged in the community (Figure 5; Welch’s *t*-test, *p* < 0.001). Even though potential biomass productivity was reduced in both groups with increasing function loss (Kruskal–Wallis, *p* < 0.001), oligotrophs were particularly robust under medium (i.e. observed) and high loss of function mutation rate. This was despite oligotrophs having a slightly greater maintenance burden than copiotrophs under the assumption that the higher affinity ABC transporters are energy dependent. Thus, there was a greater benefit for a species to invest in the (competitive) acquisition of scarce resources relative to the cost of a higher maintenance burden. An additional trait not included in the model is cell size—it has been noted that small cell sizes (or more accurately, a greater surface area-to-volume ratio) is a trait linked to genome streamlining, which would allow for even more efficient resource uptake without an energy investment (Button, 1991). Simulated oligotrophs also had an order of magnitude lower maximum growth rate. Together with efficient resource acquisition, these combined traits are thought to explain successful growth under nutrient-poor conditions (Koch, 2001; Ho et al., 2017; Finn et al., 2021). The novelty of these simulations is to demonstrate that, not only will such traits be more useful in a relatively nutrient-poor environment (i.e. bulk soil) at a given point in time, but as loss of function mutants continuously emerge in communities as a result of genome streamlining, resource scarcity will be exacerbated and oligotrophic adaptations will become even more necessary.

The rhizosphere showed a different trend with copiotrophs producing greater biomass, albeit marginally so (Figure 5, Welch’s *t*-test, *p* < 0.001). As mentioned above, fast-growing microorganisms are enriched in the rhizosphere (Li et al., 2014; Ai et al., 2015; Gkarmiri et al., 2017) and so these results are consistent with such observations. However, typically the enrichment of copiotrophs is more pronounced than in these simulations. Copiotrophs *in situ* may have additional traits to out-compete oligotrophs for a range of resources that were not included in this model. Furthermore, increasing loss of function mutants did not increase biomass production of copiotrophs (Kruskal–Wallis, *p* > 0.05). This was likely a technical effect of both the very small increase in production (Figure 4) and noise inherent with generating random communities over the 1,000 Monte Carlo simulations for each mutation rate. As copiotrophic cheaters have the highest maximum growth rate and lowest maintenance burden, *in situ* one could reasonably expect these taxa to be the most competitive. However even in the rhizosphere, there will be some limit to overall growth, dependent on root exudation and competition with the plant host for certain growth requirements, e.g. mineral nitrogen.

## Assumptions and limitations

There were a number of assumptions and limitations to the model that are worth expanding upon as these may have an impact on how loss of function could drive communities towards a Tragedy. For example, it has been suggested that copiotrophs have a higher maintenance burden than oligotrophs (Fierer et al., 2007) while in these simulations the oligotrophs had a greater burden due to an assumed greater energy demand of high-affinity ABC transporters. Comparisons of cumulative biomass production in the bulk soil and rhizosphere simulations, where copiotrophs had a greater maintenance burden than oligotrophs, did not differ (Supplementary Figure 4) and this suggests that contrasting maximum growth rate and transporter affinity were primarily responsible for observed results. Other interactions of potential importance, such as direct antagonism (e.g. antibiotic production) were not included here and could be a means for black queens to control cheater populations. A completely different evolutionary strategy could include niche differentiation of preferred carbon source, for example if one cheater taxon became specialised on growth with fatty acids, it could avoid competing with other cheaters for glucose, thus relaxing the demand on the glucose pool. Necromass was also not considered, and as microorganisms die over time, these would generate a significant alternative carbon source for cheaters (Kästner et al., 2021). Similarly, if different cheaters were auxotrophs for specific amino acids, e.g. Species 1 lacked lysine while Species 2 lacked arginine, this would relax the overall demand for each pool. Some arbuscular mycorrhizal fungi are auxotrophs for specific vitamins (Kobayashi et al., 2018) and competition between auxotrophic prokaryotes and hyphal structures could result in very different cheater dynamics, making them less successful in the rhizosphere. Finally, the addition of new genetic material from horizontal gene transfer will be a process occurring simultaneously with, and in direct competition to, genome streamlining (Vos et al., 2015). These are all biological factors likely to buffer the rate of emergence/impact of cheaters.

A number of physical factors could also reduce the negative impact of too many cheaters. The separation of cells in space is highly heterogeneous when considering total numbers within 20 μm of each other (Raynaud and Nunan, 2014) and this separation could limit the impact of any singular cheater. Soil mineralogy plays a fundamental role in adsorbing organic material and preventing microbial access (Dungait et al., 2012). This could make auxotrophy a particularly risky strategy where cheaters are not in close contact with black queens. Furthermore, these relationships could be quite different depending on the soil texture—a soil with a high clay content could be harder for cheaters to acquire needed growth factors relative to a sandy soil. All of these factors could serve to reduce the success of cheaters, slowing the frequency by which they emerge and become established in communities. Ultimately, this would put the community at less risk of a Tragedy outcome.

## Conclusion

While our model can be considered as being relatively simplistic, it nonetheless expands on theoretical concepts related to the Black Queen hypothesis and shows several robust outcomes. Specifically, these analyses move beyond two-species systems, consider two public goods simultaneously, differentiate life strategies in simulated species and consider two contrasting environments that favour distinct sets of traits. In agreement with many *in situ* observations, free-living bulk soil communities tended to benefit from co-operation and oligotrophic life strategies, while copiotrophy and cheating was rewarded in the host-associated rhizosphere. The most successful functional group was neither black queens nor cheaters, but rather those species that balanced providing an important benefit, i.e. production of the growth-limiting factor, at a relatively low personal cost, i.e., second lowest maintenance burden. Accumulation of loss of function mutants risked driving communities towards a Tragedy of the Commons where community function was dependent on co-operation between species (bulk soil) but increased biomass production where resources were externally supplied (rhizosphere). Finally, an oligotrophic life strategy improved individual growth where the loss of function risked driving the community towards a Tragedy outcome. It can be concluded that loss of function mutation is a successful evolutionary strategy in host-associated and/or resource non-limiting environments; however, cheater accumulation does pose a risk to communities that must co-operate with each other for mutual co-existence. The greater biological significance of this is that community assembly in bulk soil follows distinct rules from the rhizosphere, likely being more dependent on species possessing traits that promote co-operation under carbon and energy-limited conditions.

## Data availability statement

The original contributions presented in the study are included in the article/Supplementary material, further inquiries can be directed to the corresponding author.

## Author contributions

DF: conceptualization, coding and modelling, writing. MA: conceptualization and coding and modelling. LH: conceptualization and editing. CT: conceptualization, editing, and funding acquisition. All authors contributed to the article and approved the submitted version.

## Funding

The study was financially supported by the German Research Foundation (DFG; Project numbers 403668538 and 500452744) in context of the Priority Programmes “Rhizosphere spatiotemporal organisation—A key to rhizosphere functions” (SPP2089) and “Systems ecology of soils—energy discharge modulated by microbiome and boundary conditions” (SPP2322), and the Agency for Renewable Raw Materials (FNR Grant number 2219NR419).

## Conflict of interest

The authors declare that the research was conducted in the absence of any commercial or financial relationships that could be construed as a potential conflict of interest.

## Publisher’s note

All claims expressed in this article are solely those of the authors and do not necessarily represent those of their affiliated organizations, or those of the publisher, the editors and the reviewers. Any product that may be evaluated in this article, or claim that may be made by its manufacturer, is not guaranteed or endorsed by the publisher.
