# Supplementary material for: Reconciling concepts of black queen and tragedy of the commons in simulated bulk soil and rhizosphere prokaryote communities
Source: Front Microbiol. 2022 Sep 15;13:969784. doi: 10.3389/fmicb.2022.969784 (PMC9520196; doi:10.3389/fmicb.2022.969784)
Supplement: Supplementary file 1 [file Table_1.DOCX]

Supplementary Equations

Equation 1, change in cellulose pool over time

$\frac{dC}{dt}=C- \sum S_{c}(C_{max}\left( \frac{C}{C+C_{km}} \right))$

Equation 2, change in glucose pool over time

$\frac{dG}{dt}= \sum\alpha S_{c}\left( C_{max}\left( \frac{C}{C+C_{km}} \right) \right)- \sum S_{i}(T_{max}\left( \frac{G}{G+T_{km}} \right))$

Equation 3, change in amino acid pool over time

$$\frac{dA}{dt}= \sum S_{p}\left( \frac{A}{A+A_{km}} \right)- \sum S_{a}(T_{max}\left( \frac{A}{A+T_{km}} \right))$$

Equation 4, change in prototrophic species *p* biomass over time

$$\frac{dS_{p}}{dt}=T_{max}\left( \frac{G}{G+T_{km}} \right){S_{p}}^{\left( \frac{\mu_{max}}{Y_{glu}} \right)+(1-m)}- \gamma S_{p}$$

Equation 5, change in auxotrophic species *a* biomass over time

$$\frac{dS_{a}}{dt}=T_{max}\left( \frac{G}{G+T_{km}} \right)T_{max}\left( \frac{A}{A+T_{km}} \right){S_{a}}^{\left( \frac{\mu_{max}}{Y_{glu}} \right)+(1-m)}- \gamma S_{a}$$

Equation 6, logistic regression curve

$$y= \frac{A}{1+e^{-k (x-x_{0})}}$$

Where *A* is a parameter for the asymptote, *k* is a rate of increase over *x*, and *x_0_* is the *y* intercept.

Supplementary Table 1: Model parameters, symbols and explanations.

| Parameter | Symbol | Explanation |
| --- | --- | --- |
| Time (generations) | *t* | Each time point is expressed as a species’ generation. For simplicity, this is the same for all species. |
| Cellulose (µM) | *C* | Cellulose acts as the carbon source for the system, with 2 mM added every 200 generations. |
| Glucose (µM) | *G* | Cellulose must first be catabolised to glucose by cellulase. Glucose is subsequently taken up by species for growth. |
| Cellulase maximum activity and half-saturation constant (per µM) | *C_max_* , *C_Km_* | Cellulase activity is based on Michaelis-Menten kinetics and performs as the soil bacterium *Bacillus brevis* (Singh and Kumar, 1998). Only cellulytic species produce cellulase. |
| Glucose units per Cellulose | *α* | Glucose yield from one molecule of Cellulose. |
| Amino Acids (nM) | *A* , *A_Km_* | Amino acids are produced by Prototrophs and secreted as a leaky function based on Monod kinetics. Auxotrophs are dependent on taking up amino acids for growth. |
| Transporter maximum activity and half-saturation constants (per µM) | *T_max_* , *T_Km_* | Transporters are necessary for uptake of glucose and, in the case of auxotrophs, amino acids. T_max_ is consistent between species and is based on *E. coli* phosphotransferase (Ferenci, 1996). T_Km_ differs between copiotrophs and oligotrophs. Oligotrophs have half the Km of copiotrophs, and thus double the affinity for growth substrates. |
| Maximum growth rate and growth yield efficiency on glucose (per µM) | *µ_max_* , *Y_glu_* | The maximum growth rate of a species. Oligotrophs have an order of magnitude lower *µmax* than copiotrophs, and thus grow slower. Y_glu_ is based on optimal *E. coli* growth under continuous culture, where 57% of input glucose is converted to biomass (Kayser *et al*., 2005). |
| Maintenance energy | *m* | Maintenance energy is the burden associated with a species’ functional repertoire. The higher the *m*, the slower the overall growth rate. Cellulytic Prototrophs have the highest *m*, followed by Cellulytic Auxotrophs, Non-cellulytic Prototrophs. Non-cellulytic Auxotrophs have the lowest *m*. Oligotrophs also have a slightly higher *m* than copiotrophs due to their higher-affinity, ATP-dependent transporters. |
| Mortality (%) | *γ* | Mortality rate of each species per generation, arbitrarily set to 20%. |
| Species biomass (ng) | *S_i_* | Increase or decrease in a species’ biomass per generation is a function of the parameters that make up its functional repertoire, life strategy and the capacity for the community as a whole to produce public goods. |
| Cellulytic species biomass (ng) | *S_c_* | Those species capable of producing cellulase. |
| Prototrophic species biomass (ng) | *S_p_* | Those species capable of producing amino acids. |
| Auxotrophic species biomass (ng) | *S_a_* | Those species incapable of producing amino acids. |

Supplementary Table 2: Linear relationships between functional groups and community outcomes ascertained by linear regression in R.

| Environment | Functional group | Response variable | Beta-coeff | Std. Error | t value | *p* | Adj.R^2^ |
| --- | --- | --- | --- | --- | --- | --- | --- |
| Bulk Soil | Black Queens | Biomass | 0.16 | 0.01 | 11.48 | < 0.001 | 0.12 |
|  |  | Shannon | 0.2 | 0.02 | 12.11 | < 0.001 | 0.13 |
|  | Cellulytic Auxotrophs | Biomass | -0.24 | 0.01 | -19.11 | < 0.001 | 0.27 |
|  |  | Shannon | -0.17 | 0.02 | -10.63 | < 0.001 | 0.1 |
|  | Non-cellulytic Prototrophs | Biomass | 0.27 | 0.01 | 23.21 | < 0.001 | 0.35 |
|  |  | Shannon | 0.17 | 0.02 | 10.93 | < 0.001 | 0.11 |
|  | Cheaters | Biomass | -0.21 | 0.01 | -15.1 | < 0.001 | 0.18 |
|  |  | Shannon | -0.21 | 0.02 | -12.75 | < 0.001 | 0.14 |
| Rhizosphere | Black Queens | Biomass | -0.06 | 0.002 | -21.77 | < 0.001 | 0.32 |
|  |  | Shannon | -0.003 | 0.001 | -6.49 | < 0.001 | 0.04 |
|  | Cellulytic Auxotrophs | Biomass | -0.04 | 0.003 | -12.59 | < 0.001 | 0.14 |
|  |  | Shannon | 0.002 | 0.001 | 5.28 | < 0.001 | 0.03 |
|  | Non-cellulytic Prototrophs | Biomass | 0.04 | 0.003 | 12.2 | < 0.001 | 0.13 |
|  |  | Shannon | 0.003 | 0.001 | 7.17 | < 0.001 | 0.05 |
|  | Cheaters | Biomass | 0.07 | 0.003 | 22.44 | < 0.001 | 0.33 |
|  |  | Shannon | -0.003 | 0.004 | -6.45 | < 0.001 | 0.04 |


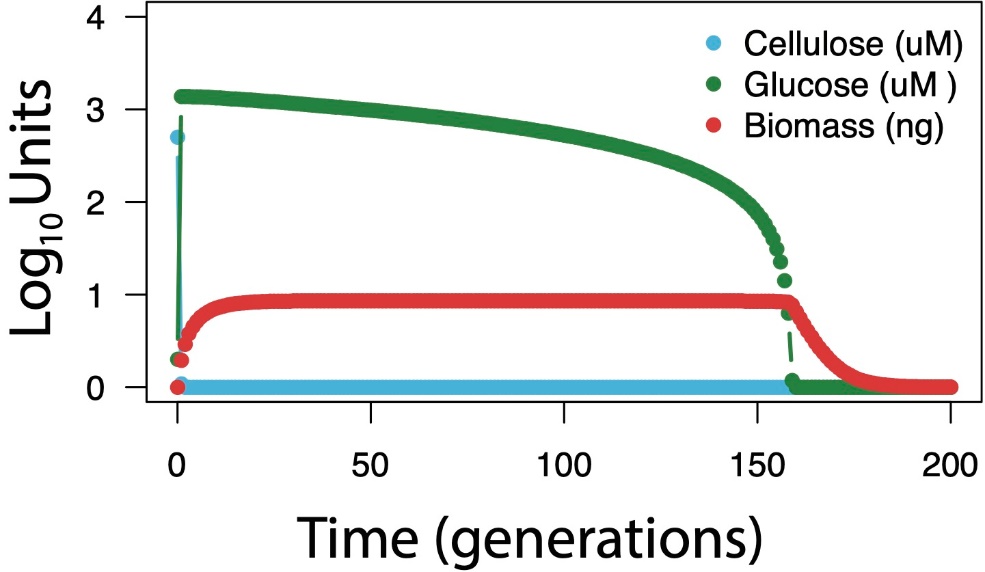


Supplementary Figure 1: Example growth curve of a copiotrophic Cellulytic Prototroph in the simulated Bulk Soil environment. Cellulose and glucose resource pools are also shown.

Supplementary Figure 2: Log_10_ cumulative biomass production and Shannon diversity index of randomly generated Bulk Soil and Rhizosphere communities.


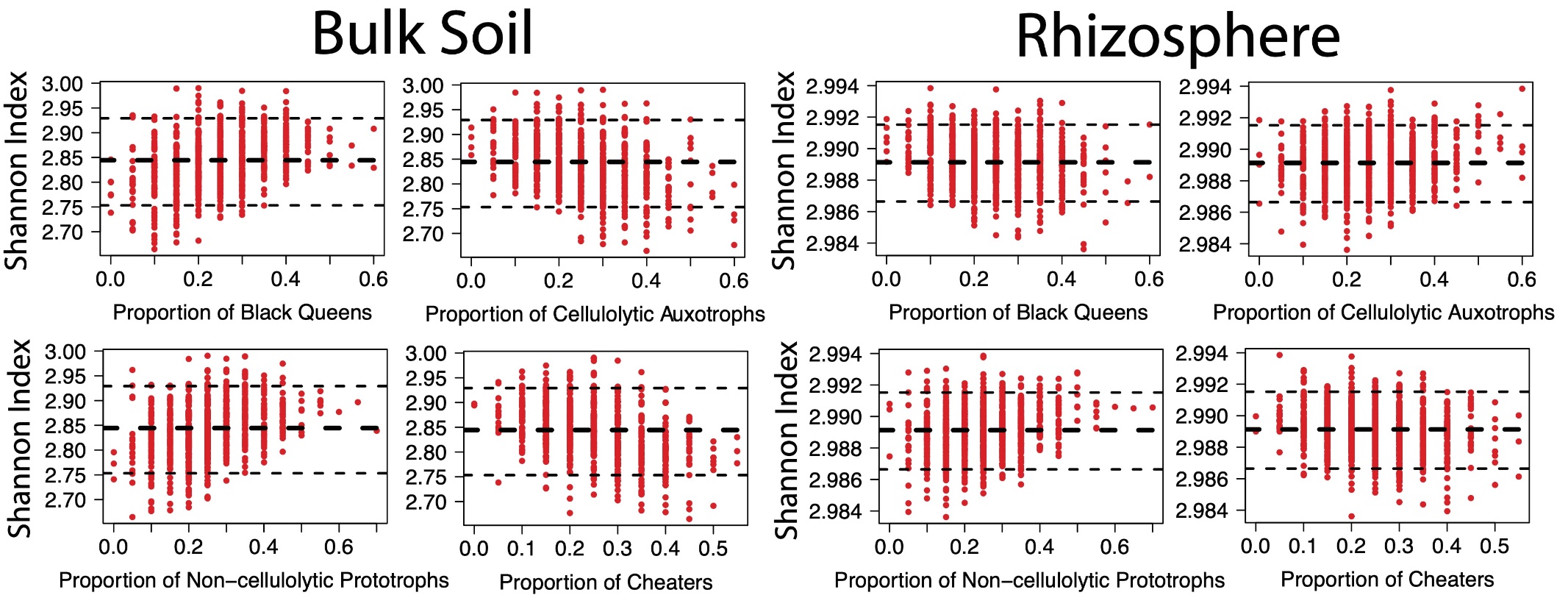


Supplementary Figure 3: Relationship between functional groups and biodiversity in simulated communities under null mutation rates. The *x* axis shows relative proportion of a given function within twenty species communities. The *y* axis shows Shannon Index over 500 generations, per simulation. The thick dotted black lines show median Shannon, while the thin dotted black lines show upper and lower 5% quantiles.


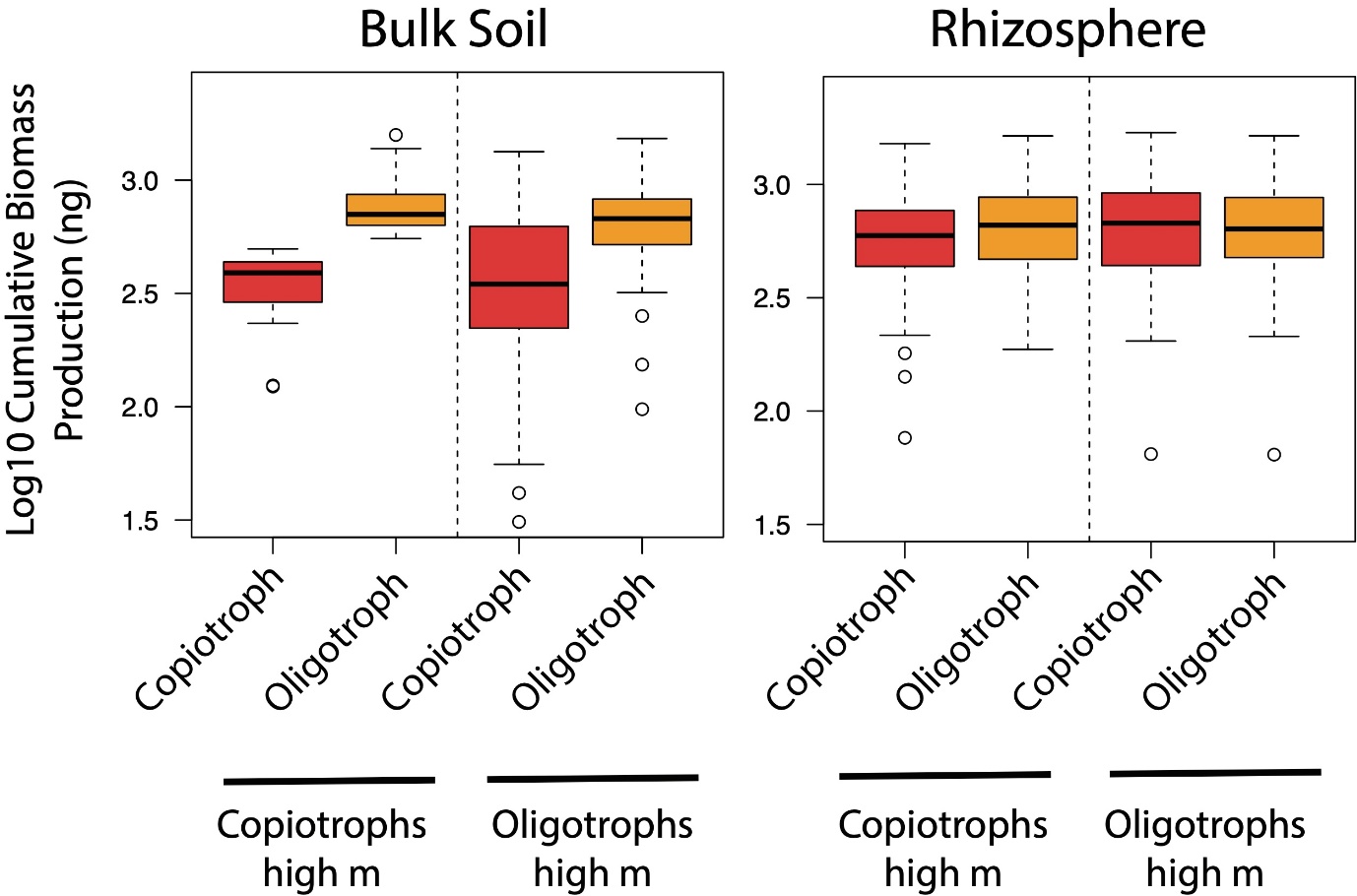


Supplementary Figure 4: Maintenance burden comparisons between copiotrophs and oligotrophs, within bulk soil and rhizosphere environments, under the null mutation rate.
